# Genetic and phylogenetic analysis of the ticks from the Sinai Massif, Egypt, and their possible role in the transmission of *Babesia behnkei*

**DOI:** 10.1007/s10493-017-0164-4

**Published:** 2017-08-28

**Authors:** Mohammed Alsarraf, Ewa J. Mierzejewska, Eman M. E. Mohallal, Jerzy M. Behnke, Anna Bajer

**Affiliations:** 1 Department of Parasitology, Institute of Zoology, Faculty of Biology, University of Warsaw, 1 Miecznikowa Street, 02-096 Warsaw, Poland; 2 Desert Research Center, Cairo, Egypt; 3 School of Life Sciences, Faculty of Medicine and Health Sciences, University of Nottingham, Nottingham, NG7 2RD UK

**Keywords:** *Hyalomma dromedarii*, *Rhipicephalus*, *Babesia*, Genotyping, Rodents, Sinai

## Abstract

Following the description of *Babesia behnkei* in the region of St. Katherine, Sinai, the present study was undertaken to determine the role of local tick species as vectors of piroplasms. First we assessed the local fauna of ticks, especially species occurring on rodents, camels and encountered in the environment, and then we compared genotypes of ticks from isolated wadis. Finally, we assessed the role of local tick species as potential vectors of *Babesia* spp. During our expedition to the Sinai Massif in a 4-week period in August–September 2012, 393 ticks were collected, including 235 adult questing ticks collected from the environment (ground level in the wadis) and 158 engorging ticks from camels and rodents. Amplification and sequencing of a 600 bp fragment of the conservative 18S rDNA and a 440 bp fragment of the more variable mitochondrial (mt) 16S rDNA were carried out to enable the identification of 54 ticks and to assess the genetic variability of ticks collected from two distant isolated wadis. The camel tick *Hyalomma dromedarii* constituted the majority (80–90%) of adult ticks collected from three wadis in the Sinai Mountains near St. Katherine. Among juvenile ticks collected from rodents, three genotypes were identified: *H. dromedarii*; *Hyalomma* sp. showing low homology with *H. dromedarii*, *H. lusitanicum* or *H. aegyptium*; and *Rhipicephalus* sp. A new genotype of *Hyalomma* was identified in an isolated montane valley, W. Gebal. *Babesia/Theileria* DNA was not detected in any of the ticks, which is likely due to the low infection rate in the limited number of ticks that were examined.

## Introduction

Hard ticks from the family Ixodidae are important ectoparasites and vectors of numerous pathogens worldwide. Ticks from subfamilies Hyalomminae and Rhipicephalinae are commonly found on livestock and domestic animals (i.e. dogs) worldwide, especially in dry hot habitats such as those of the Mediterranean and African regions. *Hyalomma* is a genus of hard-bodied ticks, common in Asia, Europe, and North Africa. These ticks exhibit great geographical and individual variability, causing problems with identification of constituent species (Apanaskevich and Horak 2010). Despite their wide geographical distribution, there are few studies on the genetic diversity of, and phylogenetic relationships between, ticks within the genus *Hyalomma* (Black et al. 1997; Black and Piesman 1994; Dobson and Barker 1999; Mangold et al. 1997). In the semi-desert montane habitats of the Sinai Massif, camels play a vital role in the transportation of goods, including food and tourists, and *H. dromedarii* is a tick species that is known to have a significant impact on camel health (Elghali and Hassan 2009). For example, it is known to be a vector of the Crimean–Congo hemorrhagic fever virus (CCHFV) (Akuffo et al. 2016; Biglari et al. 2016; Whitehouse 2004). Questing ticks of this species adopt an aggressive strategy for finding potential hosts, and even humans can be attacked by these ticks while walking along tracks frequently used by camels or while stopping and/or camping near camel rest sites. We expected this tick species to be the most prevalent in our collection of ticks from both hosts (camels, rodents) and the environment (paths in montane valleys, wadis).

Ticks are the only known vectors of piroplasms of the genus *Babesia*. In our long-term study of parasitic infections of rodents in the region, we monitored the prevalence of several haemoparasites, including *Babesia* spp. in rodents inhabiting specific ecosystems in the isolated, semi-desert montane valleys in the Sinai Massif. We have described the spatio-temporal patterns affecting the occurrence of specific parasites in spiny mouse *Acomys dimidiatus* (Alsarraf et al. 2016; Bajer et al. 2006; Behnke et al. 2004) and described a new species, *Babesia behnkei*, in Wagner’s gerbil *Dipodillus dasyurus* from Wadi (valley) Gebal (Bajer et al. 2014). Interestingly, we have detected marked differences in the distribution of ectoparasites among the four research sites (valleys), with mice from wadis Tlah and Gharaba infested by predominantly by fleas, and rodents from W. Gebal infested with juvenile ticks (Bajer et al. 2006).

Following the description of *B. behnkei*, the present study was undertaken to determine the potential role of local tick species as vectors of this piroplasm. To achieve this aim, using molecular genetic methodology, we: (1) surveyed the local fauna of ticks, especially species occurring on rodents, camels and encountered in the environment (at ground level and on rocks) in each valley/wadi; (2) compared and analyzed genotypes of ticks from the different wadis; and finally (3) assessed the role of the common tick species as vectors for *Babesia* spp.

## Methods

### Tick collection

During our expedition to the Sinai Massif in a 4-week period in August–September 2012, 393 ticks were collected, including 235 adult questing ticks from the environment (ground level and rocks in each wadi) and 158 foraging ticks from camels *Camelus dromedarius* and rodents (Table 1).

**Table 1:** The number of ticks collected by from camels, rodents and the environment by tick stage and site

Site	Adults (questing in the environment)	Adults (feeding on camels)	Larvae and nymphs (feeding on rodents)	Total
W. Arbaein	37	ND	ND	37
W. Gebal	165	ND	22	187
W. Gharaba	33	135	1	169
Total	235	135	23	393

Questing ticks were collected while walking and inspecting camel paths and resting sites in three montane valleys (wadis)—Wadi Arbaein (*n* = 37), W. Gebal (*n* = 165) and W. Gharaba (*n* = 33). Additional description of the valleys and a map of the study sites is provided in Behnke et al. (2004).

Foraging ticks from camels originated from one wadi, W. Gharaba (*n* = 135) (Table 1). Juvenile ticks (10 larvae, 11 nymphs and two juvenile individuals of an unknown stage [damaged]) were collected from live-trapped rodents from W. Gebal and W. Gharaba (Table 1). A detailed description of the rodent trapping procedures that were used, is provided in our earlier papers (Alsarraf et al. 2016; Bajer et al. 2014; Behnke et al. 2004).

### DNA extraction

Collected ticks were preserved in 70% ethanol and kept at the room temperature. Adult ticks were identified using the key by Estrada-Peña et al. (2004) before DNA extraction. All ticks from the camels and the environment were identified as a *H. dromedarii*, and among them there were 170 males and 153 females. The sex of 48 ticks could not be determined with certainty. Total DNA was extracted from individual specimens using the ‘genomic extraction’ A&A Biotechnology kit (Poland) following the manufacturer’s instructions. Adult ticks were cut in half along the longitudinal axis to provide the optimal weight of tissue sample recommended by the manufacturer. Total DNA was eluted in 160 μl of elution buffer. Extracted DNA was stored in −20 °C for further procedures.

### Genotyping and phylogenetic analysis of ticks

Two genetic markers were used; in the first step, amplification of a 600 bp fragment of the conservative 18S rDNA was carried out following the method by Noureddine et al. (2011), and after assessment of the quality of the extracted DNA used for identification of the species of tick. In the second step, to assess the genetic variability of ticks between two distant isolated wadis (W. Gebal and W. Gharaba) the more variable mitochondrial (mt) 16S rDNA (440 bp) was amplified (Kulakova et al. 2014).

Unfortunately, extraction of informative DNA from ethanol-fixed ticks was successful for only 54 specimens, including 38 adult ticks (all identified as *H. dromedarii* by morphological features) and 16 juvenile ticks from rodents (9 nymphs, 6 larvae, 1 unidentified stage) (Table 2).

**Table 2:** The DNA isolates of ticks by the genes studied, site where hosts were sampled and species of host

Host/environment	W. Gebal		W. Gharaba	
	18S rDNA	mt 16S rRNA	18S rDNA	mt 16S rRNA
Camel	ND	ND	5	9
Rodents				
*A. dimidiatus*	7	4	ND	ND
*A. russatus*	6	ND	ND	ND
*D. dasyurus*	1	1	ND	ND
*S. calurus*	1	ND	1	ND
Environment	3	27	ND	ND
Total	18	32	6	9

#### Analysis of 600-bp 18S rDNA

For the amplification of the 18S rDNA, the specific primers used were: 18S-F1, 5′-AACCTGGTTGATCCTGCCAGTA-3′ and 18S-R1, 5′-TAGCGCCGCAATACGAATGC-3′, following the PCR protocol by Noureddine et al. (2011). Twenty-four amplicons (Table 2) were sequenced by a private company (Genomed, Poland). The sequences we obtained were aligned using MEGA v. 6.0. (Tamura et al. 2013) and compared with sequences deposited in the GenBank database (BLAST NCBI). Representative sequences have been deposited in GenBank (Table 3).

**Fig. 1:**
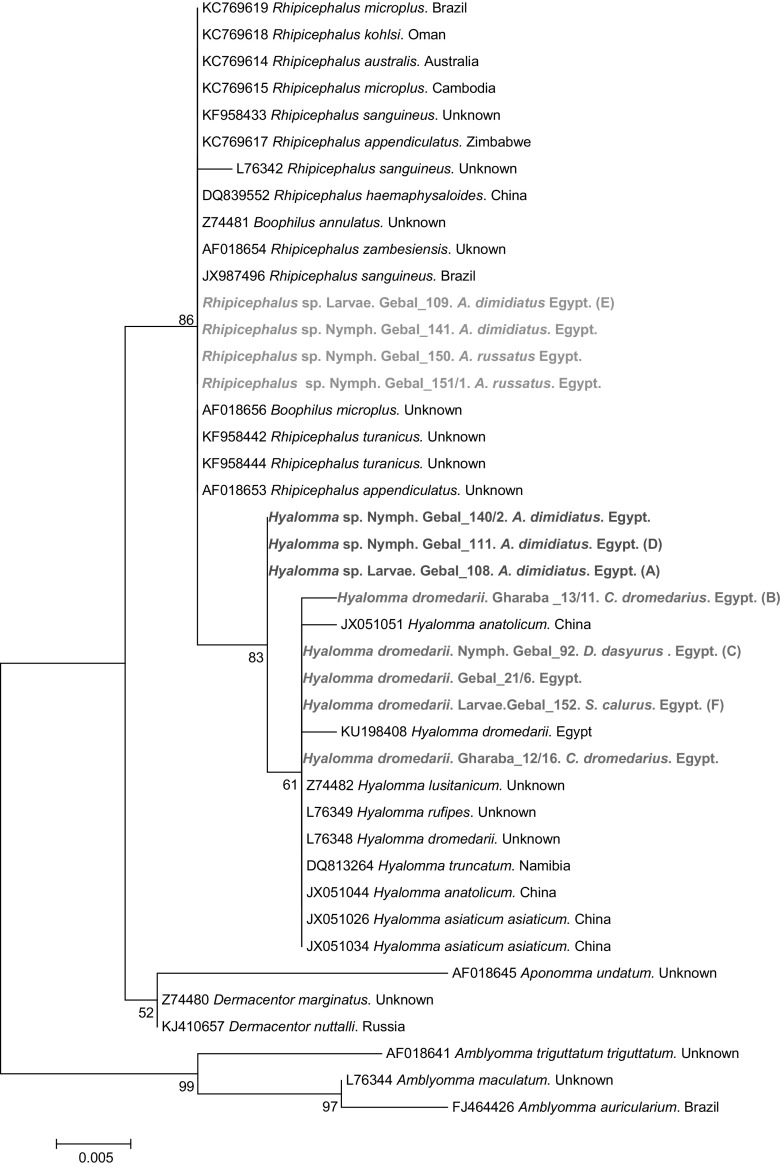
The phylogenetic tree of ticks based on a fragment of the 18S rDNA gene, was inferred using the maximum likelihood method and Kimura 2-parameter model with Gamma distribution. The percentage of replicate trees in which the associated taxa clustered together in the bootstrap test (1000 replicates) are shown next to the branches. The analysis involved 42 nucleotide sequences. All positions containing gaps and missing data were eliminated. Evolutionary analyses were conducted in MEGA6.0

For the phylogenetic analysis, the Akaike information criterion was used in jModel Test to identify the most appropriate model of nucleotide substitution. A representative tree for 18S rDNA was constructed using MEGA v. 6.0, by the Maximum Likelihood method and Kimura 2-parameter model with Gamma distribution (Fig. 1).

#### Analysis of 440-bp mt 16S rDNA

Amplification of a 440 bp fragment of mt16S rDNA was performed with the specific primers: 16SF, 5′-TTGCTGTGGTATTTTGACTA-3′ and 16SR, 5′-CCGGTCTGAACTCAGATC-3′, following the PCR protocol described previously by Kulakova et al. (2014). Forty-two amplicons were sequenced, 32 from W. Gebal, 9 from W. Gharaba (Table 2) and one isolate from an adult questing tick from W. Arbaein (Table 3) by a private company (Genomed). The resulting sequences were aligned using MEGA v. 6.0. (Tamura et al. 2013) and compared with sequences deposited in the GenBank database (BLAST NCBI). Representative sequences have been deposited in GenBank (Table 3).

**Table 3:** Origin of the ticks isolates used for genotyping and phylogenetic analyses

Gene	Isolate number	Isolate code	Group	GenBank accession number	Tick stage	Tick host/environment	Site (wadi)
18S rDNA	108	A	G2/MT1	KY512792	Larva	*A. dimidiatus*	Gebal
	13/11	B	G1/MT2	KY512790	Adult	*C. dromedarius*	Gharaba
	92	C	G1/MT2	KY512791	Larva	*A. dimidiatus*	Gebal
	111	D	G2/MT1	KY512794	Nymph	*A. dimidiatus*	Gebal
	109	E	G3/MT3	KY512793	Larva	*A. dimidiatus*	Gebal
	152	F	G1	KY512795	Larva	*S. calurus*	Gebal
mt 16S rDNA	108	A	G2/MT1	KY512799	Larva	*A. dimidiatus*	Gebal
	13/11	B	G1/MT2	KY512796	Adult	*C. dromedarius*	Gharaba
	92	C	G1/MT2	KY512798	Larva	*A. dimidiatus*	Gebal
	111	D	G2/MT1	KY512800	Nymph	*A. dimidiatus*	Gebal
	109	E	G3/MT3	KY512801	Larva	*A. dimidiatus*	Gebal
	15/3	G	MT2	KY512797	Adult	Environment	Arbaein

**Fig. 2:**
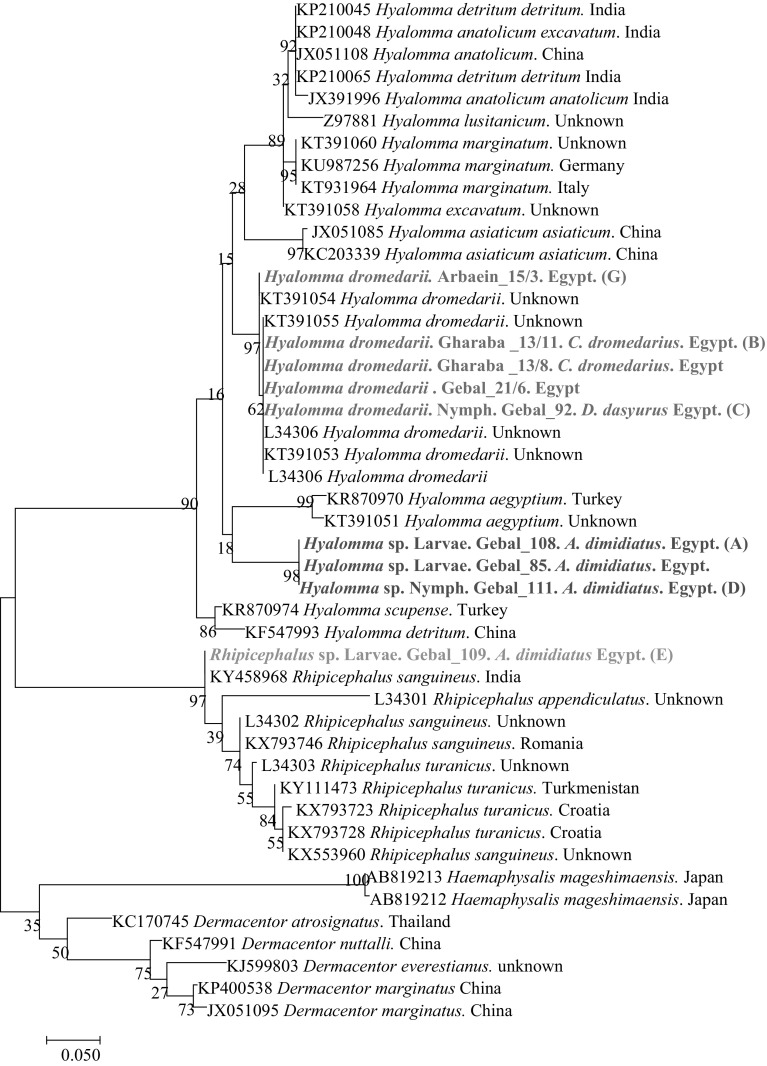
The phylogenetic tree of ticks, based on a fragment of the mt 16s rDNA gene, was inferred using the maximum likelihood method and a General Time Reversible model with Gamma distribution. The percentage of replicate trees in which the associated taxa clustered together in the bootstrap test (1000 replicates) are shown next to the branches. The analysis involved 46 nucleotide sequences. All positions containing gaps and missing data were eliminated. Evolutionary analyses were conducted in MEGA6.0

For the phylogenetic analysis, the Akaike information criterion was used in jModel Test to identify the most appropriate model of nucleotide substitution. A representative tree for mt16S rDNA was constructed using MEGA v. 6.0, by the Maximum Likelihood method and General Time Reversible model with Gamma distribution (Fig. 2).

### Detection of *Babesia* spp. in ticks

Detection of *Babesia* was performed by nested PCR amplification of the 18S rRNA gene (Bajer et al. 2014). The primers and thermal profiles used in this study have been described previously (Matjila et al. 2008; Nijhof et al. 2003; Oosthuizen et al. 2008). Reactions were performed in 1 × PCR buffer, 1 U Taq polymerase, 1 μM of each primer and 2–5 μl of the extracted DNA sample. Negative controls were performed in the absence of template DNA. Genomic DNA of *B. microti* King’s Collage strain was used as positive control.

Primers Nbab1F 5′-AGCCATGCATGTCTAAGTATAAGCTTTT-3′ (Oosthuizen et al. 2008) and TB Rev 5′-AATAATTCACCGGATCACTCG-3′ (Matjila et al. 2008) were used in the first round of the PCR for the amplification of a 1700 bp near-full-length sequence from the 18S rRNA gene. Primers GF 5′-G(C/T)(C/T)TTGTAATTGGAATGATGG-3′ and GR 5′-CCAAAGACTTTGATTTCTCTC-3′ were then used in the second round to amplify the 559 bp fragment of 18S rDNA (Bonnet et al. 2007). This method was previously successfully applied for the detection of *B. behnkei* in rodents (Bajer et al. 2014).

## Results

### Local tick fauna

All the questing adult ticks collected from the three wadis that were sampled (Table 1) were identified as *H. dromedarii* based on morphological features. This species constituted the majority of ticks collected from the camels (80–90%). The remaining ticks were identified as *Rhipicephalus* sp. based on morphological features.

Juvenile ticks collected from rodents were engorged, preventing accurate identification by morphological feature, but 16 were successfully genotyped (Table 5). Eight ticks (5 nymphs, 2 larvae, 1 unidentified damaged specimen) were identified as *Rhipicephalus* sp. and eight as *Hyalomma* spp., including two *H. dromedarii* (1 nymph and 1 larvae) and six *Hyalomma* sp. (3 nymphs, 3 larvae) (Figs. 1, 2; Table 5).

### Genetic diversity of ticks

We successfully amplified DNA from 54 ticks, including 38 adults, 9 nymphs, 6 larvae and one unidentified juvenile tick from a rodent (damaged). For 12 of these 54 ticks both genetic markers, the conservative 18S rDNA and the more variable mt16S rDNA, were successfully amplified and sequenced. For the remaining ticks only one sequence was obtained and analyzed (Table 2).

#### Analysis of the 600-bp fragment of the 18S rDNA

Alignment of 24 sequences, including 18 sequences obtained from diverse groups of ticks from W. Gebal (3 questing adult *H. dromedarii* and 15 juvenile ticks from rodents) and five sequences from ticks collected from camels and one juvenile tick from a rodent in W. Gharaba (all ticks identified as *H. dromedarii*), revealed three distinct groups of genotypes (Fig. 1). However, a BLAST search did not allowed clear, unambiguous identification of to tick species level, because all of our sequences showed very high similarity (99.19–100%) to the reference sequences of three tick species, *H. dromedarii*, *H. lusitanicum* and *R. sanguineus* (accession numbers L76348, Z74482 and JX987496, respectively).

The phylogenetic analyses of the 18S rDNA sequences of the representative isolates described in this work (Tables 2, 3) and the reference isolates from the GenBank database revealed the presence of four major groups (Fig. 1). Our isolates clustered in three subgroups in the first major group (Fig. 1). The first of our subgroups (G1) contained 10 isolates, five of them are shown in the phylogenetic tree in Fig. 1. Sequences from this subgroup clustered together with the *H. dromedarii* reference sequence (L76348) and were separated from *H. rufipes* and *H. lusitanicum* (Fig. 1). The second of our subgroups (G2) contained six isolates from juvenile ticks collected from rodents in W. Gebal (Table 5), and three of these are illustrated in the phylogenetic tree (Fig. 1). These sequences were separated from both subgroups G1 (*H. dromedarii*) and G3 (*Rhipicephalus* sp.), and there is no reference sequence in GenBank that corresponds to this subgroup. Our third subgroup (G3) contained eight isolates from juvenile ticks collected from rodents in W. Gebal (Table 5), and four of these are incorporated in the phylogenetic tree (Fig. 1). This subgroup formed a sister branch with a branch grouping several documented *Rhipicephalus* species recorded in GenBank (Fig. 1), so we identified it primary as *Rhipicephalus* sp., despite the lack of reference sequence in GenBank for our specific branch of this subgroup.

#### Analysis of 440-bp mt16S rDNA

Alignment of 42 sequences, including 33 sequences obtained from diverse groups of ticks from W. Gebal (28 questing adult *H.dromedarii* and 5 juvenile ticks from rodents) and nine sequences from ticks collected from camels in W. Gharaba (all ticks identified as *H. dromedarii*), revealed three distinct groups of genotypes (Fig. 2).

The first group of genotypes, MT1, contained three isolates which were identical to each other. One of these (isolate A- sequenced from a larval tick collected from a spiny mouse in W. Gebal) is shown in Tables 3 and 4, and all are included in Fig. 2. A BLAST search for the genotype MT1 revealed highest similarity to the reference sequence of *H. dromedarii* (L34306; 91.06% [316/347 nucleotides]), and lower similarity (89.94%) to *H. lusitanicum* (Z97881) and *H. aegyptium* (88.68%; KT391051) (Table 4).

**Table 4:** Similarity of mt 16 rDNA sequences of ticks from Sinai Massif with the most similar references sequences from the GenBank database

	*Hyalomma lusitanicum* Z97881	*Hyalomma detritum* KF547993	*Hyalomma aegyptium* KT391051	*Hyalomma* sp. (MT1) Isolate B	*Hyalomma dromedarii* (MT2) Isolate E	*Hyalomma dromedarii* (MT2) Isolate G	*Hyalomma dromedarii* (MT2) Isolate A	*Rhipicephalus* sp. (MT3) Isolate C
*Hyalomma dromedarii* L34306	315/347 90.77%	311/345 90.14%	312/347 89.91%	316/347 91.06%	343/344 99.70%	342/344 99.41%	342/343 99.70%	290/349 83.09%
*Hyalomma lusitanicum*Z97881		303/347 87.31%	305/345 88.40%	313/348 89.94%	314/347 90.84%	315/347 90.77%	313/346 90.46%	288/348 82.75%
*Hyalomma detritum* KF547993			302/345 87.53%	305/345 88.40%	310/345 89.85%	311/345 90.14%	309/344 89.82%	297/353 84.13%
*Hyalomma aegyptium* KT391051				306/345 88.69%	312/347 89.91%	313/347 90.20%	301/331 90.93%	289/349 82.80
*Hyalomma* sp. (MT1) Isolate B					316/346 91.32%	317/346 91.61%	315/345 91.30%	289/349 82.80
*Hyalomma dromedarii* (MT2) Isolate E						342/343 99.70%	342/342 100%	290/348 83.33%
*Hyalomma dromedarii* (MT2) Isolate G							341/342 99.70%	291/348 83.62%
*Hyalomma dromedarii* (MT2) Isolate A								289/347 83.28%

The second group of genotypes, MT2, contained 38 isolates (Table 5), with a similarity between them of 99.70–100% (Table 4). Three representative isolates (B, C and G) are shown in Tables 3 and 4 and five are incorporated in Fig. 2. A BLAST search for MT2 revealed highest similarity to *H. dromedarii* (L34306; 99.41–99.70%) and a much lower similarity (90.46–90.84%) to *H. lusitanicum* (Z97881) (Table 4). The similarity of genotype MT2 to genotype MT1 was in the range 91.30–91.61% (Table 4). A homology of >99% with *H. dromedarii* supports the conclusion that these isolates were indeed *H. dromedarii*, in agreement with the outcome of the analysis of 18S rDNA (G1 group) and identification based on morphological features.

**Table 5:**
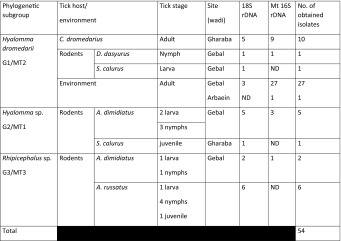
Classification of tick isolates to genetic groups based on combined results of 18S rDNA and mt 16S rDNA typing (ND- not done)

The third subgroup MT3, contained one isolate (E) from a tick larva collected from *A. dimidiatus* in W. Gebal (classified in G3 group of 18S rDNA isolates). The isolate H is shown in Tables 3, 4 and 5 and included in Fig. 2. This genotype, MT3, showed the highest similarity to *H. detritum* (KF547993; 84.13%) and *H. dromedarii* (L34306; 83.09%) and a clear-cut identification of the tick species was not possible based on mt 16S rDNA because there was no available reference sequence of *Rhipicephalus/Boophilus* in the GenBank database (Table 4). The MT3 genotype displayed also a relatively low homology with genotypes MT1 (82.80%) and MT2 (*H. dromedarii*; 83.28–83.62%), a difference of more than 50 nucleotides in 350 bp gene fragment (Table 4).

The phylogenetic analysis of the mt 16S rRNA sequences of our isolates/genotypes and available reference sequences from the GenBank database revealed the presence of three major groups (Fig. 2). The first group contained eight representative sequences, grouping together genotypes MT1 and MT2, which formed two subgroups in the *Hyalomma* genus. The first subgroup (MT1 genotype) contained three sequences from larvae and nymphs from rodents from W. Gebal, illustrated on the phylogenetic tree in Fig. 2 as unnamed *Hyalomma* sp. These sequences clustered together in one branch, located between the *H. aegyptium* and *H. dromedarii* subgroups (Fig. 2). The second subgroup (MT2 genotypes) clustered with *H. dromedarii* references sequences from the GenBank database.

The second major group contained one sequence from a larvae from *A. dimidiatus* from W. Gebal (genotype MT3). This isolate clustered with different sequences of *Rhipicephalus* genus and was identified as *Rhipicephalus* sp. based on phylogenetic analysis of 18S rDNA (Fig. 1). The MT3 sequence formed as branch of its own, separated clearly from all other sequences and located between the *H. dromedarii* and *Haemaphysalis megashimaensis* subgroups (Fig. 2). The third major group contained sequences from the GenBank database of two other genera of ticks, *Dermacentor* and *Haemaphysalis* sequences (Fig. 2).

### Detection of *Babesia* spp. in ticks

No *Babesia*-positive samples were detected by nested PCR in 54 specimens, including 38 adult *H. dromedarii*/*Hyalomma* sp. ticks and 16 juvenile ticks from rodents (8 *Hyalomma* spp., 8 *Rhipicephalus* sp.).

## Discussion

In the present study, we have confirmed that the camel tick, *H. dromedarii* constitutes the main tick hazard for camels, local people and tourists visiting the Sinai Mountains in the St. Katherine area. We have demonstrated that the tick community of rodents is diverse, with new genetic variants of *Hyalomma* and *Rhiphicephalus* sp. recognized in isolated montane wadis. Despite our examination of ticks collected from rodents in W. Gebal, where a new species of *Babesia* was described recently from Wagner’s gerbil, no *Babesia* DNA was detected in ticks.

The camel tick *H. dromedarii* constituted the majority of almost 400 ticks collected from three wadis in the Sinai Mountains near St. Katherine, including questing adults from the environment and feeding individuals from camels. This finding underlines the need for the improved control of tick infestation on camels, as these are the main hosts for both adults and nymphal ticks. Despite the efforts of local veterinary services, infestation of camels is still high and ticks were found attacking humans on camel tracks and in their resting sites.

In order to determine the vector of *B. behnkei*, we tried to collect also juvenile ticks from rodents inhabiting four wadis. Despite the examination of 345 rodents (Alsarraf et al. 2016; Bajer et al. 2014), juvenile ticks were found attached to only a small number of rodents from two wadis: W. Gebal localized at the highest attitude (Behnke et al. 2004) and a single juvenile tick on a mouse from W. Gharaba. Thus, at this time of the year (late summer) infestation of rodents with juvenile ticks was very low. However, molecular and phylogenetic analyses revealed that the genetic diversity of ticks from rodents is high, and three distinct genetic groups were identified: *H. dromedarii*, *Hyalomma* sp. and *Rhipicephalus* sp. Interestingly, analysis of both genetic markers supported the separation of several *Hyalomma* isolates from rodents from W. Gebal from *H. dromedarii* and other *Hyalomma* species (*H. lusitanicum*, *H. aegyptium*). This may suggest evolution/differentiation of new tick genotypes based on spatial isolation, as W. Gebal is a site localized among high mountains (ab. 2000 m a.s.l.), subject to severe climatic conditions, accessible with camels and only on foot, and characterized by a lack of permanent inhabitants. Interestingly, the new *Babesia* species, *B. behnkei* was highly prevalent among Wagner’s gerbils in this wadi, and absent or very rare in the remaining three less isolated montane wadis (Bajer et al. 2014).

Analyses of both genetic markers allowed the separation of the third group of tick isolates from rodents (group G3 and MT3). These isolates were identified as *Rhipicephalus* sp., based on the closest BLAST match of 18S rDNA with *R. sanguineus* (437/437 identical nucleotides) and the localization of the MT3 sequence between the *H. dromedarii* and *Haemaphysalis* subgroups. However, the lack of a reference *Rhipicephalus* sequences in GenBank for mt 16S rDNA prevented more precise identification of this tick isolate.

In the present study we were unsuccessful in identifying the vector of *B. behnkei*, despite examination of ticks from rodents from the exact location where the piroplasm was originally isolated from an infected Wagner’s gerbil (W. Gebal), and this included a tick from one Wagner’s gerbil which we found to be positive for *B. behnkei* DNA. However, it is well known from numerous studies carried on *Ixodes ricinus* ticks in Europe (Stańczak et al. 2015; Welc-Falęciak et al. 2012), that the prevalence of *Babesia* spp. in ticks may be extremely low, often in the range 1–5%, thus the number of ticks that we were able to incorporate in our study in Sinai may have been too low to detect the presence of *Babesia*. For this reason further studies on ticks from this wadi are required before the genotypes that we have described herein can be eliminated as vectors. Interestingly, despite the low prevalence of *Babesia* in ticks, prevalence of this parasite in rodent hosts can be high, reaching 40–60% (Bajer et al. 2014; Tołkacz et al. 2017; Welc-Falęciak et al. 2008), possibly because of the recently demonstrated vertical transmission of piroplasms in rodents (Bednarska et al. 2015; Tołkacz et al. 2017). The possibility of vertical transmission of *B. behnkei* in *D. dasyurus* therefore also requires further study.

## Conclusions

In this paper we have demonstrated that *H. dromedarii* constitutes the main component of the tick fauna in the Sinai Massif of Egypt. We have also provided evidence for genetic variability of ticks of the genus *Hyalomma* between isolated wadis, resulting most likely from their geographic isolation. No ticks positive for *Babesia* were found, suggesting other routes of transmission between rodents.
